# Supplementary material for: Characterisation of Plasmodium vivax lactate dehydrogenase dynamics in P. vivax infections
Source: Commun Biol. 2024 Mar 22;7:355. doi: 10.1038/s42003-024-05956-6 (PMC10959993; doi:10.1038/s42003-024-05956-6)
Supplement: Supplementary file 2 — Supplementary information [file 42003_2024_5956_MOESM2_ESM.pdf]

Supplementary Information for "Characterisation of Plasmodium vivax lactate dehydrogenase dynamics in P. vivax infections"

Pengxing Cao[1,*], Steven Kho[2,3,*], Matthew J. Grigg[2], Bridget E. Barber[4], Kim A. Piera[2], Timothy William[2], Jeanne R. Poespoprodjo[3,5], Ihn Kyung Jang[6], Julie A. Simpson[7], James M. McCaw[1,7], Nicholas M. Anstey[2,#], James S. McCarthy[4,8,#,†], Sumudu Britton[4,#,†]

1. School of Mathematics and Statistics, University of Melbourne, Melbourne, Australia
2. Global and Tropical Health Division, Menzies School of Health Research and Charles Darwin University, Darwin, Australia
3. Papuan Community Health and Development Foundation, Timika, Papua, Indonesia
4. QIMR Berghofer Medical Research Institute, Brisbane, Australia
5. Department of Pediatrics, Timika General Hospital, Timika, Papua, Indonesia
6. Diagnostics Program, PATH, Seattle, USA.
7. Centre for Epidemiology and Biostatistics, Melbourne School of Population and Global Health, University of Melbourne, Melbourne, Australia
8. Department of Infectious Diseases, Melbourne Medical School, Melbourne, Australia

* Equal contribution
\# Equal contribution
† Correspondence to James S. McCarthy (james.mccarthy@unimelb.edu.au) and Sumudu Britton (sumudu.britton@health.qld.gov.au)

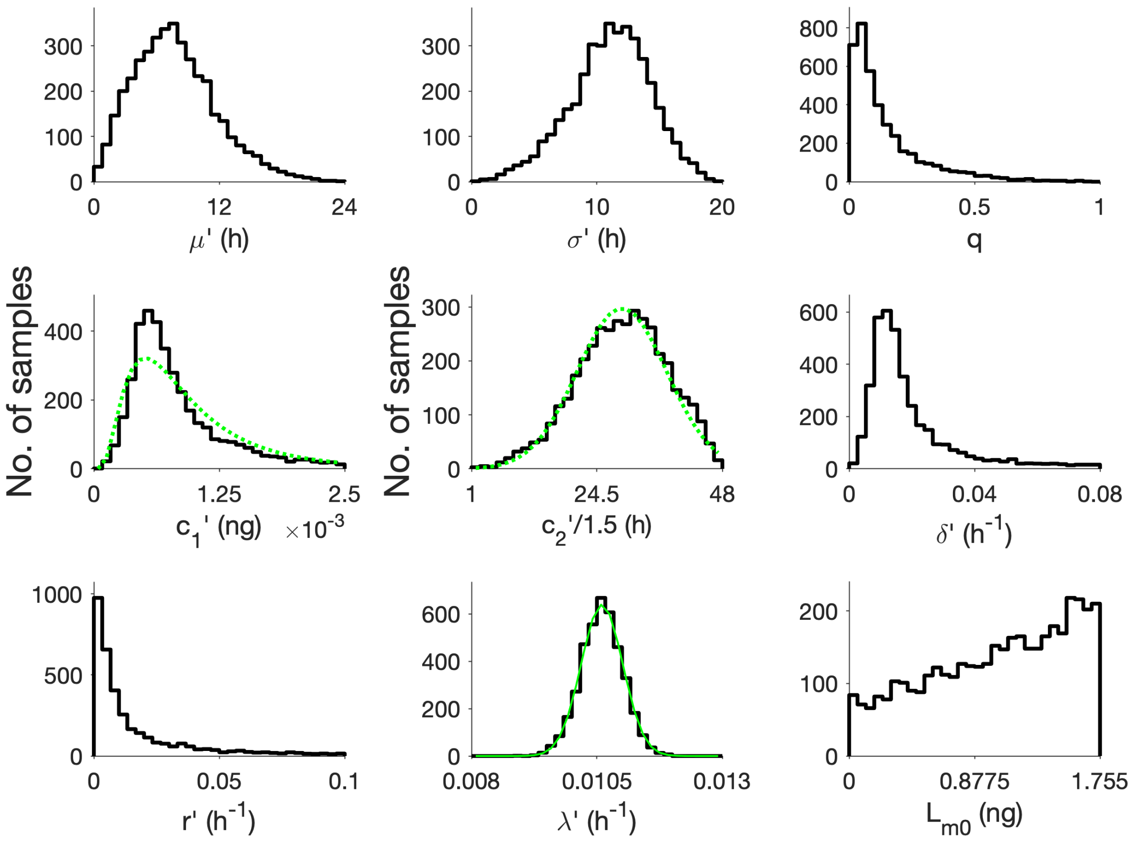

Figure S1: Posterior distributions of the population mean parameters of the ex vivo model. The prior distributions for the model parameters are uniform distributions specified in Table 1 in the main text except that the prior for the *ex vivo* PvLDH decay rate λ' is a bounded normal distribution shown by the green curve (and also specified in Table 1). The marginal posterior distributions of c_1' and c_2' were empirically fitted by a lognormal distribution logN(-7.15, 0.65) and a normal distribution N(29.17, 8.40), respectively, shown by the green dotted curves. The empirical fits were used as prior distributions for c_1 and c_2 in the within-host model fitting (see Table 2 in the main text). Note that the range of c_2' was scaled down by 1.5 such that its empirical fit is applicable to the within-host model fitting.

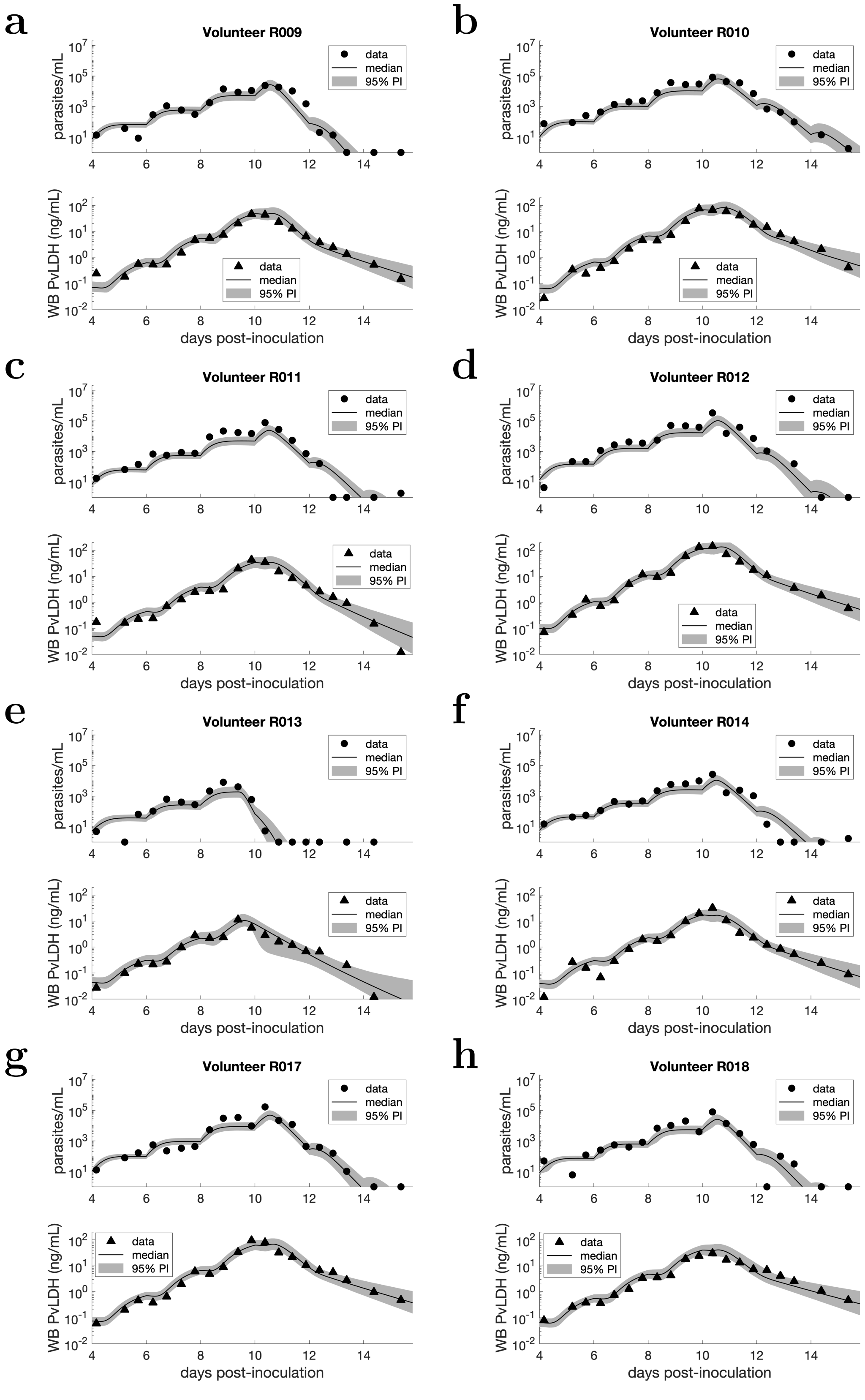

Figure S2: Data and model fits for eight VIS volunteers. Solid dots are clinical data and model fits are shown by the median and 95% PI of the model-predicted distributions of parasitemia and whole blood PvLDH concentration (in ng per mL blood) at different time points.

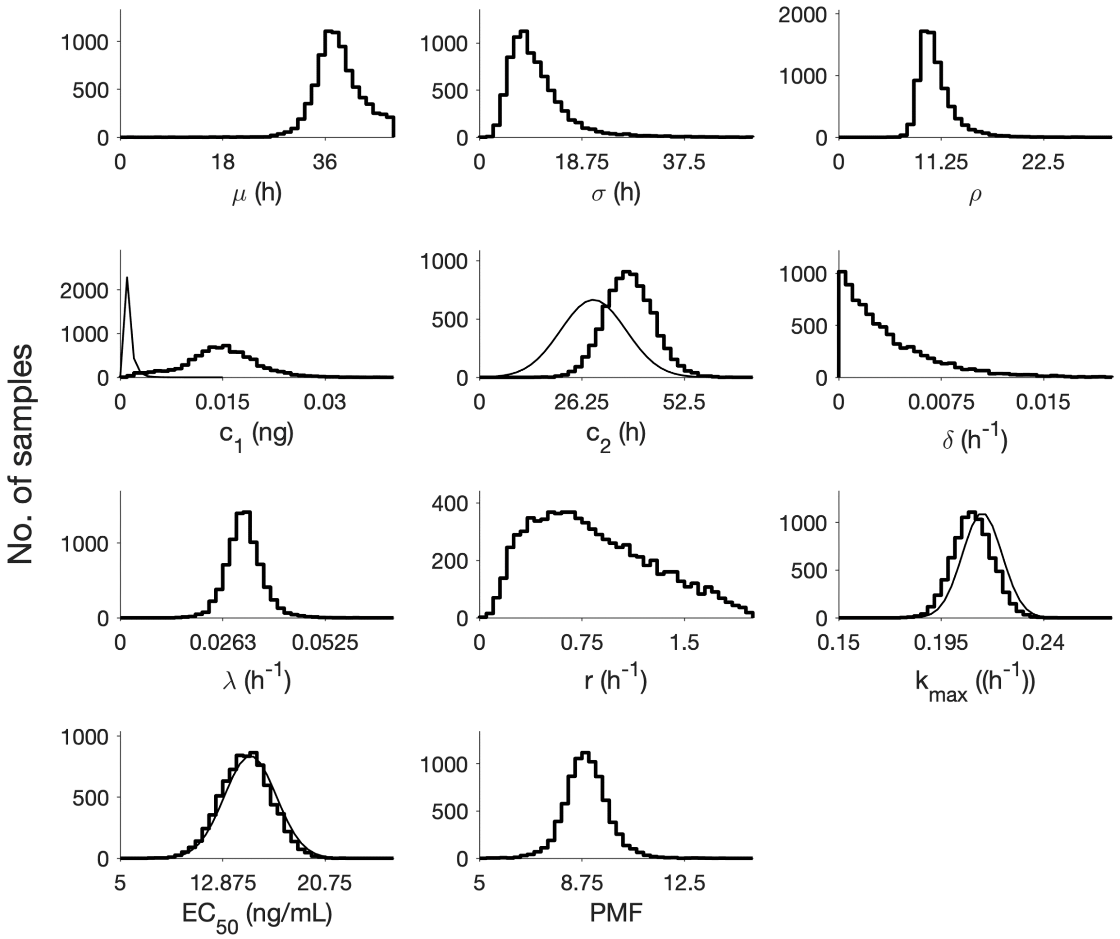

Figure S3: Posterior distributions of the population mean parameters of the within-host model. The prior distributions for the model parameters are either uniform distributions or bounded normal/lognormal distributions, as specified in Table 2. Normal/lognormal priors are shown by the smooth curves. The distribution of the parasite multiplication factor (PMF), which is calculated based on the posterior samples of ρ and δ and $PMF = \rho e^{-\delta a_L}$, is shown in the last panel.

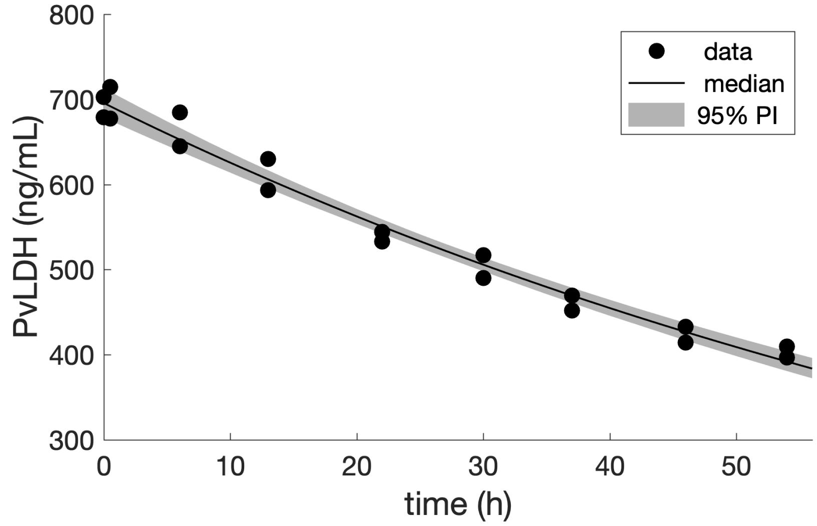

Figure S4: Result of fitting the exponential decay model (Eq. 1 in the main text) to the *in vitro* decay measurements of human PvLDH (see the Materials and Methods for further details). Black dots are experimental data, and experimental measurements were conducted in duplicate at each timepoint. The black curve is the median model prediction based on the 4,000 posterior samples. The shaded region represents the 95% prediction interval whose upper and lower boundaries are the 2.5% and 97.5% quantiles of model predicted PvLDH level at various time points.

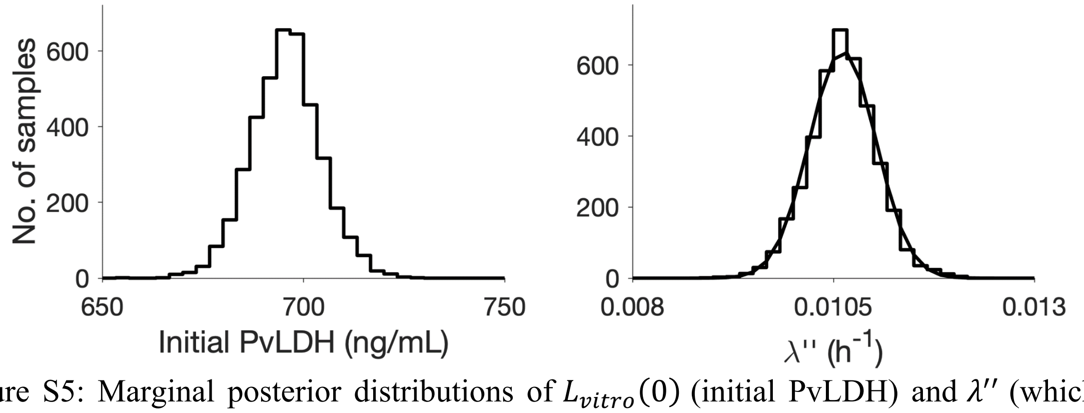

Figure S5: Marginal posterior distributions of $L_{vitro}(0)$ (initial PvLDH) and λ'' (which are parameters of the exponential decay model in Eq. 1 in the main text). The prior distributions of $L_{vitro}(0)$ and λ'' are uniform distributions U(500, 1000) and U(0, 0.1) respectively. Each of the histograms contains 4000 posterior samples. The histogram of λ'' was fitted by a normal distribution N(0.0106, 4.1565×10^{-4}) which is shown by the smooth curve. The empirical distribution of λ'', N(0.0106, 4.1565×10^{-4}), will be used as the prior distribution of the *ex vivo* PvLDH decay rate λ' in the *ex vivo* model fitting.

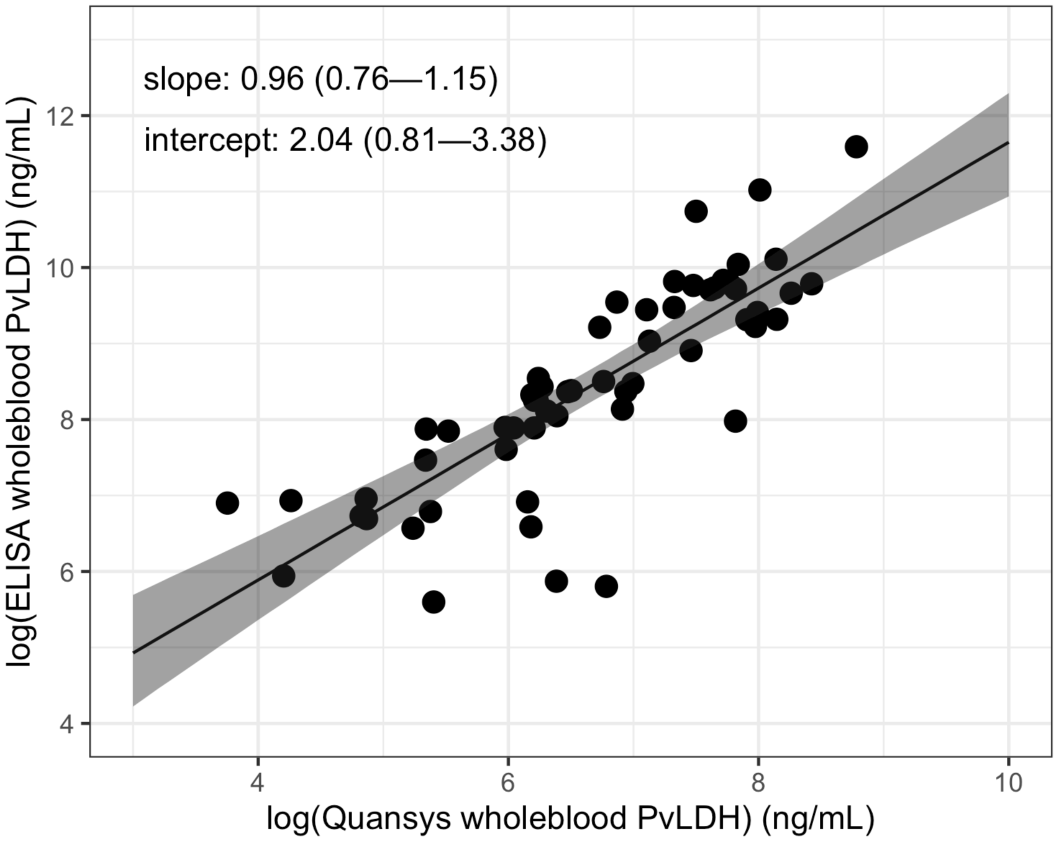

Figure S6: The relationship between Quansys measurement of whole blood PvLDH and inhouse ELISA measurement of whole blood PvLDH. Experimental data (black dots) are obtained from 58 Malaysian patients whose whole blood PvLDH was measured by both methods. The data are log-transformed and fitted by a linear model. The median and 95% prediction interval are shown by the black line and the shaded area, respectively. The median estimates and 95% credible intervals (shown in parentheses) of the slope and intercept parameters of the linear model are also provided in the figure.
